# Genome editing for vegetable crop improvement: Challenges and future prospects

**DOI:** 10.3389/fgene.2022.1037091

**Published:** 2022-11-22

**Authors:** Ruma Devi, Shivani Chauhan, Tarsem Singh Dhillon

**Affiliations:** Department of Vegetable Science, Punjab Agricultural University, Ludhiana, India

**Keywords:** genome-editing technology, CRISPR-cas application, vegetable crops, advanced, cutting-edge

## Abstract

Vegetable crops are known as protective foods due to their potential role in a balanced human diet, especially for vegetarians as they are a rich source of vitamins and minerals along with dietary fibers. Many biotic and abiotic stresses threaten the crop growth, yield and quality of these crops. These crops are annual, biennial and perennial in breeding behavior. Traditional breeding strategies pose many challenges in improving economic crop traits. As in most of the cases the large number of backcrosses and stringent selection pressure is required for the introgression of the useful traits into the germplasm, which is time and labour-intensive process. Plant scientists have improved economic traits like yield, quality, biotic stress resistance, abiotic stress tolerance, and improved nutritional quality of crops more precisely and accurately through the use of the revolutionary breeding method known as clustered regularly interspaced short palindromic repeats (CRISPR)-CRISPR-associated protein-9 (Cas9). The high mutation efficiency, less off-target consequences and simplicity of this technique has made it possible to attain novel germplasm resources through gene-directed mutation. It facilitates mutagenic response even in complicated genomes which are difficult to breed using traditional approaches. The revelation of functions of important genes with the advancement of whole-genome sequencing has facilitated the CRISPR-Cas9 editing to mutate the desired target genes. This technology speeds up the creation of new germplasm resources having better agro-economical traits. This review entails a detailed description of CRISPR-Cas9 gene editing technology along with its potential applications in olericulture, challenges faced and future prospects.

## Introduction

The world population is estimated to increase by 10 billion in the next three decades, thereby the demand for food crops is likely to increase by 25–70% (Hunter et al., 2017). Contemporary agriculture will eventually face enormous challenges to produce crops having high yield and better quality which require few inputs (Tilman et al., 2011). Vegetable crops act as protective foods that provide essential nutrients in the human diet due to their richness in vitamins, minerals, dietary fiber and phytochemicals (Dias, 2012). More than 400 g of fruits and vegetables should be consumed daily per person to reduce the risk of cardiovascular diseases. (Bazzano et al., 2002). or cancer (Aune et al., 2017). Like any other food crop, vegetable crops too are prone to many biotic and abiotic stresses (Jaganathan et al., 2018; Boscaiu and Fita 2020) thereby necessitating the development of next-generation architectured crops which are able to sustain adverse environmental stresses (Karkute et al., 2017).

Till now conventional breeding techniques have been extensively used to improve the yield and agronomic performance which is a complex, time-consuming and labor-intensive process (Zhang et al., 2018). It is a selection of improved individuals utilizing genetic variation in the population (Breseghello and Coelho, 2013). It also recombines the desired gene pools resulting in new genotypes or cultivars (Holme et al., 2019). However, when the desirable genetic variation is not available in the gene pool, then it can be generated and used for selection by inducing mutations using various mutagenic agents like non-ionizing radiation i.e. UV rays or ionizing radiation i.e. X and gamma rays, alpha and beta rays, fast and slow neutrons. Some chemicals can also be used as mutagenic agents like ethyl methane sulphonate (EMS), methyl methane sulphonate (MMS), hydrogen fluoride (HF), sodium azide, *N*-methyl-*N*-nitrosourea (MNU) and hydroxylamine) (Parry et al., 2009). Mutation breeding has not been extensively utilized in vegetable crop improvement except for a few exceptions being low in its efficiency. Over the last few decades, there have been a large number of significant developments in the molecular biology approaches to improve crop yield and quality. Recently, tremendous progress has been made in genome editing tools like site-directed nucleases (SDNs) which are able to edit the crops at high speed and possess great potential in shaping up the novel genetic makeup of vegetable crops (Tian et al., 2021). Genome editing tools can precisely engineer the genes by either deleting, replacing or inserting specific sequences at the specific targeted location in the target genome to generate novel traits (Corte et al., 2019). Zinc-finger nucleases (ZFNs), transcription activator-like effector nucleases (TALENs), and clustered regularly interspaced short palindromic repeats (CRISPR)/CRISPR-associated enzymes are the tools for genome editing used to modify plants (Miller et al., 2007). Site-specific double-stranded breaks (DSBs) are enabled by Crispr/Cas which further activate the cellular DNA repair systems (Zhu et al., 2020; Gaj et al., 2013). These DSBs can either be corrected by the non-homologous end joining (NHEJ) pathway or through the homology-directed repair (HDR) pathway (O’Driscoll and Jeggo, 2006; Wang T. et al., 2019a). The use of first-generation technologies like ZFNs and TALENs has been limited due to their adverse mutagenic outcome, low editing efficiency, time-consuming process and labor-intensive selection and screening process (Gaj et al., 2013; Jaganathan et al., 2018). The second-generation genome editing technology i. e. CRISPR/Cas9 is easier to design, and execute and more cost-effective. The use of CRISPR/Cas9 in vegetable crops has substantially expanded gene editing technology and made it possible to create novel genotypes with desired phenotypic features and altered genomic functions at the base pair level (Abdallah et al., 2015; Nunez de Caceres Gonzalez and De la Mora Franco, 2020). We will first go over CRISPR/Cas9 history and development before summarising how it is currently used to modify vegetable crops. Finally, we will talk about the real-world challenges in enhancing vegetable crops with the desired traits.

### Clustered regularly interspaced short palindromic repeats-CRISPR-associated protein-9

**FIGURE 1:**
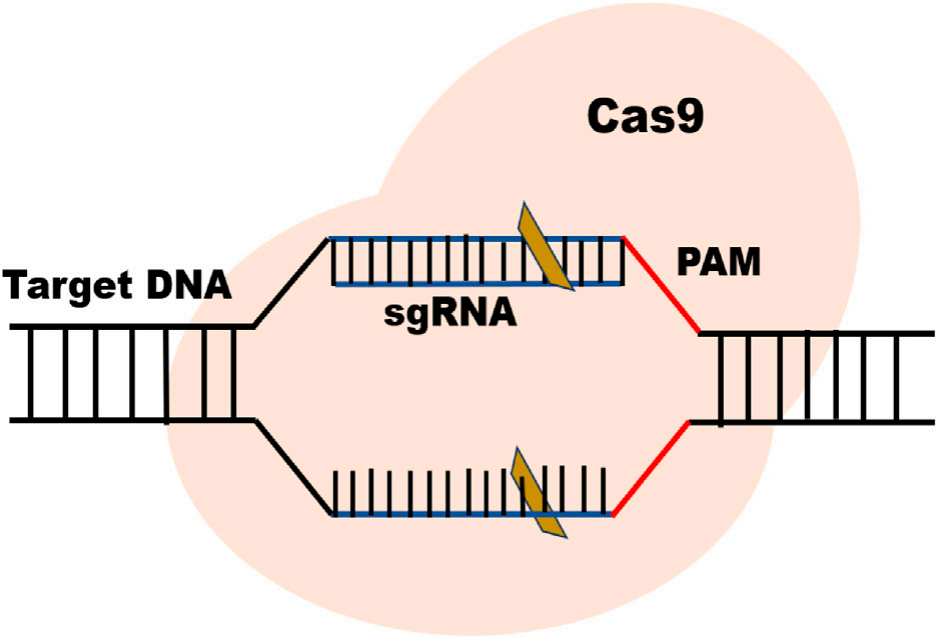
Basic structure of CRISPR/Cas9 system.

CRISPR-Cas9 is an advanced genome editing technique that enables scientists to change, add, or remove specific DNA sequences to modify specific regions of the genome. In general, there are three main types (I-III) of CRISPR-Cas systems utilized for target interference (Rouillon et al., 2013). Type II uses its two distinctive nuclease domains, RuvC and HNH, to achieve interference with only a basic effector-module design (Gasiunas et al., 2012). Type II Cas9 from *Streptococcus* pyogenes (SpCas9) is the most popular CRISPR nuclease employed in CRISPR-Cas technology (Doudna and Charpentier, 2014). The protospacer adjacent motif (PAM) is recognized by the sgRNA-Cas complex, and Cas9 cleaves the target DNA to create a double-strand break (DSB), activating cellular DNA repair processes (Figure 1).

### How does it work

**FIGURE 2:**
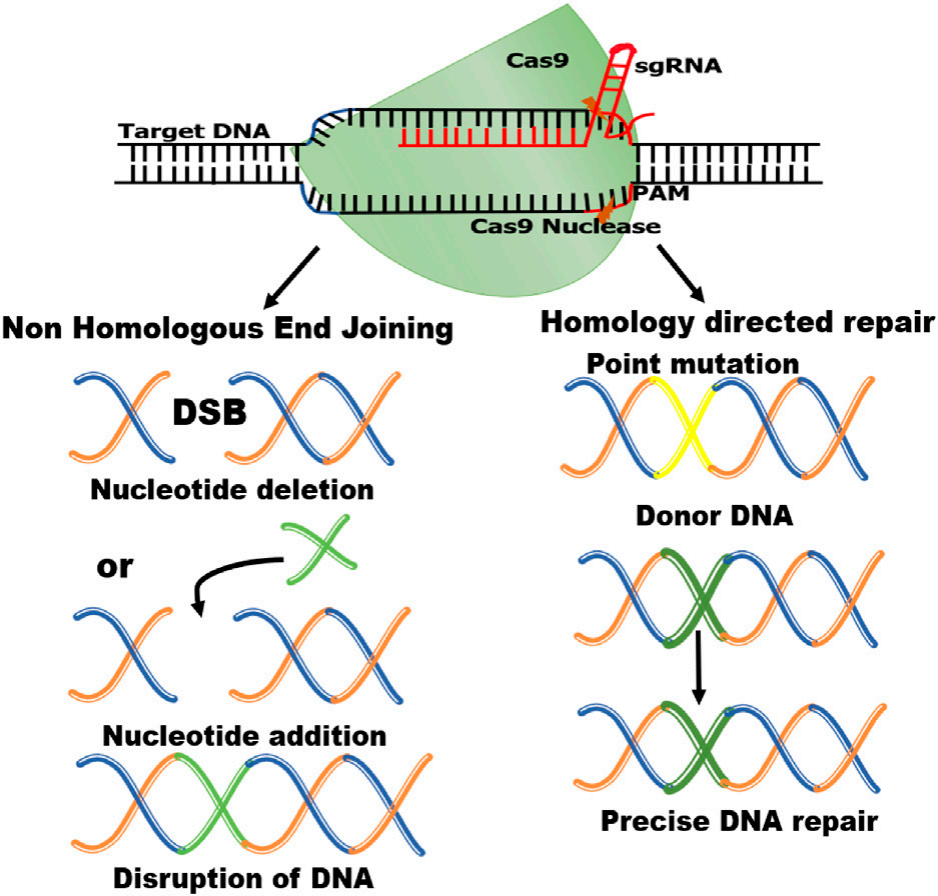
Schematic diagram of CRISPR/Cas9 mechanism. Double-strand breaks (DSBs) are induced when Cas9 enzyme and gRNA bind with targeted double-stranded DNA. These DSBs are repaired by Homology-directed repair (HDR) and Non-homologous end-joining (NHEJ) mechanisms.

The two essential parts of the CRISPR-Cas9 system that modify DNA are Cas 9 enzyme and guiding RNA (gRNA). The “genetic scissors” known as Cas9 (enzyme), cut the two DNA strands at an exact place in the genome to allow for the addition or deletion of DNA fragments and the guiding RNA (gRNA) is made up of two parts: Crispr RNA (crRNA), a 17–20 nucleotide sequence complementary to the target DNA, and a transactivating Crispr RNA (tracr RNA), which serves as a binding platform for the Cas9 nuclease (Mei et al., 2016) (Figure 1). Each crRNA hybridizes with tracr RNA, and these two RNAs jointly make a complex with the Cas9 nuclease (Deltcheva et al., 2011). The goal of the guide RNA is to find and bind to a target DNA sequence that is complementary to its RNA bases. Double strand breaks, or DSBs, are created when the Cas9 enzyme cuts across both DNA strands at the same location in the DNA sequence as the guide RNA (Jinek et al., 2012; Jiang et al., 2013). The DSBs inflicted by Cas-9 protein are repaired by two mechanisms i.e., non-homologous end-joining (NHEJ) and homology-directed repair (HDR) (Liu et al., 2019) (Figure 2). NHEJ needs enzymes in the repair mechanism in which different DNA segments are joined by excluding a homologous DNA template (Shuman and Glickman, 2007). It is an extraordinarily effective cell repair mechanism that is most often exploited but is prone to errors that can cause minor, spontaneous insertions or deletions. (Yang et al., 2020). The HDR however is quite precise in gene insertion or replacement of DNA segments at the predicted DSB site but requires a large amount of homologous DNA template (Liu et al., 2019; Yang et al., 2020).

### Modifications in clustered regularly interspaced short palindromic repeats genome editing system

Several new CRISPR systems have been developed to overcome the limitations of CRISPR/Cas 9 and improve its specificity for more effective genome editing. These cutting-edge technologies are detailed below and could be crucial resources for molecular crop breeding.

Cas protein Cpf1 (also known as Cas12a) is with RNA-guided system being widely used in genome editing (Kim et al., 2017; Moon et al., 2018; Chen et al., 2019). It uses a T-rich PAM sequence to identify the target site, extending the editing sites beyond the G-rich PAM sequences preferred by Cas9. The target site of Cpf1 is located at the distal position and downward the PAM sequence. Cpf1’s guide RNA has 43 base pairs and is shorter than the sgRNA of Cas9 (about 100 bp) (Kim et al., 2017; Chen et al., 2019). Cpf1 generates staggered-ended double-strand breaks, which offers more advantages than Cas9 and improves the effectiveness of the NHEJ-based gene insertion. (Kim et al., 2017; Moon et al., 2018). Cpf1-based genome editing has been reported in rice and soybean (Kim et al., 2017; Xu et al., 2017). Additional research is required to analyze the specificity of Cpf1 in other crops and to enhance the current Cas12a-based applications Schindele and Puchta (2020).

Cas12b prefers T-rich PAM, produces staggered double-strand breaks, and needs both a crRNA and a trans-activating crRNA. Cas12b protein is smaller than Cas9 and Cas12a. Cas12b/C2c1 has been effectively used to carry out multiplex genome editing, induce mutations, and cause deletions at many loci in Arabidopsis (Wu et al., 2020). However, Cas12b shows its optimum activity at higher temperature (Teng et al., 2018), it needs to be altered for making it more useful in crop applications.

Cas13 is a recently identified CRISPR effector which targets specific RNAs in plant cells. This system has high RNA target specificity and efficiency. Cas13 protein belongs to class 2 type VI and contains unique higher eukaryotes and prokaryotes nucleotide-binding domains that are exclusively associated with RNase activity (Wolter and Puchta 2018). Till now, three different Cas13 protein classes, such as Cas13a, Cas13b, and Cas13d, have been used for RNA editing in plants (Schindele et al., 2019), mainly to target RNA for cleavage, for combating RNA viruses (Aman et al., 2018; Wolter and Puchta 2018). It has been demonstrated that CRISPR/LshCas13a system is used to create potyvirus resistance in plants, which suggests that this system can be employed for agricultural and biotechnological applications (Aman et al., 2018).

Cas14a is a highly compact protein, which can be used as an RNA-guided DNA nuclease for target-specific single-stranded DNA (ssDNA) cleavage (Harrington et al., 2018; Khan M. Z. et al., 2019b). Due to its sequence-independent and unrestricted cleavage, it has evolved into an excellent tool for building resistance to economically significant plant ssDNA viruses (Harrington et al., 2018; Khan M. S. S. et al., 2019). Cas14a is only one-third the size of Cas9 and is the smallest working CRISPR system to date. (Harrington et al., 2018). It has the potential to generate resistance against ssDNA viruses that belong to the Geminiviridae and Nanoviridae families (Khan M. S. S. et al., 2019a).

Besides these systems, base editing provides effective, concise, and well-recognized strategies for specific base replacement at the target site without the need for DSBs or donor DNA and independent of homology-directed repair (HDR) (Chen et al., 2019). They are helpful when there is a requirement for desired protein-coding genes to create variations with enhanced economic traits (Li H. et al., 2020c). At present, there are two types of base editors: Cytosine base editors (CBE) (which converts C-G pair to T-A pair) and adenine base editors (ABE) (converts A-T base pair to G-C base pairs (Komor et al., 2016; Gaudelli et al., 2017) Figures 3A,B. Both these editors largely depend upon the availability of PAM sequence as they use DNA binding proteins to induce point mutations at the targeted site (Jin et al., 2019). The major limitation of base editors is their inability to generate precise base edits in point mutations. Research is ongoing to improve the efficiency and role of base editing in random and targeted mutagenesis (Li H. et al., 2020c).

**FIGURE 3:**
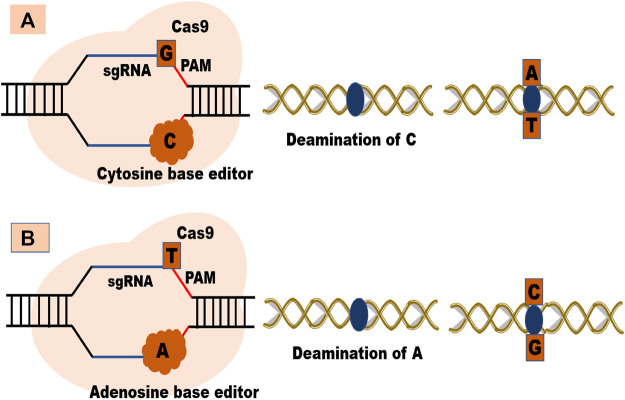
**(A)** Cytosine base editor and **(B)** Adenosine base editor.

Prime editing, a recent genome-editing tool that induced all kinds of mutations and base substitutions (insertion/deletion) without donor DNA or double-strand breaks. Prime editing uses proteins fused to Cas 9 nickase and prime edited guide RNA with reverse transcriptase to induce mutations. The pegRNA contains not only complimentary sequence as the target sites that directs nCas9 to its target sequence, but also an additional sequence creating desired sequence changes. The Cas9 nicked the PAM containing DNA strand which act as a primer for reverse transcriptase to create extensions in the nicked strand using pegRNA as template and ultimately modifying the target site (Anzalone et al., 2020). PE has been successfully applied in rice and wheat to generate stable edited lines with price gene edits (Butt et al., 2020; Li H. et al., 2020c). It is advantageous over other genome editing tools in crop science with respect to reduced off-target mutations and less requirement of PAM sequences due to RNA template and high-efficiency rates but still experiments are need to be conducted regarding its specificity and potential. The various tools and Databases used for CRISPR/Cas9 mediated genome editing in plant systems are being presented in Tables 1, 2.

**TABLE 1:** List of available tools used for CRISPR/Cas9 genome editing.

S.No.	Tool name	Purpose	Access link	References
1)	GuideMaker	To identify target genes and design sgRNA sequences	https://academic.oup.com/gigascience/article/doi/10.1093/gigascience/giac007/6562533	Poudel et al. (2022)
2)	CROPSR	For complex (polyploid) genome wide CRISPR gRNA design	https://bmcbioinformatics.biomedcentral.com/articles/10.1186/s12859-022-04593-2	Paul et al. (2022)
3)	BE target	To design sgRNA for base editing in plants	https://www.sciencedirect.com/science/article/pii/S2001037022003269	Xie et al. (2022)
4)	BE-Designer and BE-Analyzer	To design target sites and assess mutation ratios	https://europepmc.org/article/med/33180295	Hwang and Bae (2021)
5)	PnB Designer	A web application to design prime and base editor guide RNAs for animals and plants	https://bmcbioinformatics.biomedcentral.com/counter/pdf/10.1186/s12859-021-04034-6.pdf	Siegner et al. (2021)
6)	crisprRdesign	To design sgRNAs	https://www.jgenomics.com/v08p0062.htm	Beeber and Chain, (2020)
7)	CRISPR-Local	Designing sgRNAs	https://academic.oup.com/bioinformatics/article/35/14/2501/5221013	Sun et al. (2019)
8)	CRISPR-P 2.0	For computer-aided sgRNA designing to minimize off-targets	https://www.cell.com/molecular-plant/pdf/S1674-2052(17)30004-7.pdf	Liu et al. (2017)
9)	GuideScan	Designing CRISPR guide RNA libraries	https://www.ncbi.nlm.nih.gov/pmc/articles/PMC5607865/	Perez et al. (2017)
10)	Breaking-Cas	To facilitate sgRNA design	http://bioinfogp.cnb.csic.es/tools/breakingcas	Oliveros et al. (2016)
11)	CHOPCHOP v2	To increase target range and specificity	https://academic.oup.com/nar/article/44/W1/W272/2499370	Labun et al. (2016)
12)	CRISPOR	Helps to design and evaluate guide sequences	http://crispor.tefor.net/	Haeussler et al. (2016)
13)	CRISPRscan	Find gRNAs on genes, gRNA generation and scoring of gRNAs	https://pubmed.ncbi.nlm.nih.gov/26322839/	Moreno-Mateos et al. (2015)
14)	CRISPR Multitargeter	To find common and Unique sgRNA	https://www.ncbi.nlm.nih.gov/pmc/articles/PMC4351176/	Prykhozhij et al. (2015)
15)	Off-Spotter	To design optimum sgRNA	https://www.ncbi.nlm.nih.gov/pmc/articles/PMC4326336/	Pliatsika and Rigoutsos (2015)
16)	Wu-CRISPR	To design sgRNA	https://genomebiology.biomedcentral.com/articles/10.1186/s13059-015-0784-0	Wong et al. (2015)
17)	SgRNA designer (CRISPRPick)	For effective selection of SgRNA	https://portals.broadinstitute.org/gpp/public/analysis-tools/sgrna-design	Doench et al. (2014)
18)	E-CRISP	To identify target site	http://www.e-crisp.org/E-CRISP/aboutpage.html	Heigwer et al. (2014)
19)	CRISPRseek	To find potential gRNA	https://bioconductor.org/packages/release/bioc/html/CRISPRseek.html	Zhu et al. (2014)
20)	Cas-OFFinder	For identifying off-target sites	http://www.rgenome.net/cas-offinder/	Bae et al. (2014)
21)	CRISPRdirect	For selecting targets based on input sequence	https://pubmed.ncbi.nlm.nih.gov/25414360/	Naito et al. (2014)

**TABLE 2:** List of available databases for CRISPR/Cas9 for plants.

	Database name	Purpose	Access link	References
1)	CRISPR Plant v2	For highly specific sgRNAs	https://www.ncbi.nlm.nih.gov/pmc/articles/PMC6330547/	Minkenberg et al. (2019)
2)	PGED (Plant Genome Editing Database)	Stores information about mutants	http://plantcrispr.org/cgi-bin/crispr/index.cgi	Zheng et al. (2019)
3)	CRISPRlnc	Manual database of sgRNAs	https://www.crisprlnc.org/	Chen et al. (2019)
4)	Cpf1- Database	Design tool for Cpf1	http://www.rgenome.net/cpf1-database/	Park and Bae (2017)
5)	Cas-Database	Design tool for Cas9 nucleases	http://www.rgenome.net/cas-database/	Park et al. (2016)
6)	CrisprGE	CRISPR/Cas-Central repository	http://crdd.osdd.net/servers/crisprge/	Kaur et al. (2015)

## Applications of clustered regularly interspaced short palindromic repeats/CRISPR-associated protein-9 in vegetable crops

As vegetables are susceptible to various abiotic and biotic stresses which reduce optimum production, which highlights the significance of developing resistant/tolerant cultivars. Additionally, in vegetable crops, various quality traits including flavor and nutritional profile, plant architecture, and shelf life can be improved. Various gene editing systems have good potential to improve the quality and yield of vegetables among which CRISPR/Cas is very popular. The major applications of CRISPR/Cas9 in vegetable crops is being discussed below under different sections and the list of various traits modified by CRISPR/Cas is being presented in Table 3.

**TABLE 3:** List of traits modified by Crispr/Cas in vegetable crops.

Crop	Target gene	Trait modification	References
Tomato			
	*SlAMS*	Affects pollen viability	Bao et al. (2022)
	*SlHyPRP1*	Salt tolerance	Tran et al. (2021)
	*SlPelo* and *SlMlo1*	Tomato yellow leaf curl virus (TYLCV) and Powdery mildew fungus	Pramanik et al. (2021)
	*CCD8*	Host resistance to plants	Bari et al. (2021)
	*K transporter HKT 1,2*	Salt tolerance	Vu et al. (2020)
	*SlMAPK3*	Enhances heat stress tolerance	Yu et al. (2019)
	*SlMAPK3*	Drought stress response	Wang et al. (2017)
	*BZR 1*	Regulates heat stress tolerance	Yin et al. (2018)
	*Coat protein, Replicase from Tomato yellow leaf curl virus*	Induced resistance to tomato yellow leaf curl virus	Tashkandi et al. (2018)
	*SlMlo1*	Resistance to powdery mildew	Nekrasov et al. (2017)
	*Pectate lyase (PL), polygalacturonase 2a (PG2a* and *beta-galactanase (TBG4)*	Development of cell wall and modification of fruit color and weight	Wang et al. (2018a)
	*APETALA2a (AP2a), NON-RIPENING (NOR) and FRUITFULL (FUL1/TDR4* and *FUL2/MBP7)*	Development and ripening of fruit	Wang et al. (2018b)
	*SlGAI*	Gibberellin responsive dwarf mutant	Tomlison et al. (2019)
	*SBPase*	Induced Leaf senescence in SBPase mutants	Ding et al. (2018)
	*Psy1* and *CrtR-b2*	Change in Carotenoid biosynthesis	D’Ambrosio et al. (2018)
	*lncRNA1459*	Alters fruit ripening, lycopene, ethylene and carotenoid biosynthesis	Li et al. (2018a)
	*SGR1, Blc, LCY-E, LCY-B1, LCY-B2*	Increased lycopene content	Li et al. (2018b)
	*SlCBF*	The sharp decrease in chilling stress tolerance	Li et al. (2018c)
	*PDS* and *GABA-TP1, GABA-TP2, GABA-TP3, CAT9* and *SSADH*	Increased γ-aminobutyric acid content	Li et al. (2018d)
	*SlNPR1*	Reduced drought tolerance	Li. et al. (2019)
	*SlMYB12*	Pink fruit color	Deng et al. (2018)
	*RIN*	Fruit ripening	Jung et al. (2018)
	*SP, MUILT, FAS, CyCb, OVUTE* and *FW2.2*	Fruit size and lycopene accumulation	Zsogon et al. (2018)
	*SP, SP5, CLV3* and *WUS, GGP1*	Plant structure, Fruit ripening, Day-length response, Vitamin-C and fruit size	Li et al. (2018e)
	*RIN* and *ethylene*	Fruit ripening	Li et al. (2020a)
	*RIN*	Ethylene production and fruit ripening	Ito et al. (2017)
	*SlORRM4*	Fruit ripening	Yang et al. (2017)
	*Alc*	Shelf life	Yu et al. (2017)
	*CLAVATA-WUSICHEL*	Altered locule number	Rodriguez-Leal et al. (2017)
	*Solyc12g038510*	Jointless mutant, abscission	Roldan et al. (2017)
	*phytoene synthase (PSY)*	Fruit color	Filler et al. (2017)
	*LEAFYCOTYLEDON1-LIKE4*	Fruit metabolism	Gago et al. (2017)
	*SlIAA9*	Parthenocarpy	Ueta et al. (2017)
	*SlAGL6*	Parthenocarpic fruits	Klap et al. (2017)
	*ANT1*	Anthocyanin biosynthesis	Cermak et al. (2015)
	*SlALS1*	Enhanced herbicide resistance	Danilo et al. (2019)
*ALS*	Herbicide resistance	Veillet et al. (2019)
Brinjal			
	*SmelPPO4, SmelPPO5, and SmelPPO6* genes	Reduced levels of flesh browning	Maioli et al. (2020)
Potato			
	*StDND1*, *StCHL1*, and *DMG400000582*(*StDMR6-1*)	Late blight resistance	Kieu et al. (2021)
	*GBSS* genes	Starch biosynthesis	Andersson et al. (2018)
	*GBSS1*	Starch biosynthesis	Kusano et al. (2018)
	*GBSS*	Starch and tuber quality	Andersson et al. (2017)
	*St16DOX*	Glycoalkaloids metabolism	Nakayasu et al. (2018)
	*Coilin* gene	Biotic (PVY) and abiotic stress resistance	Makhotenko et al. (2019)
Carrot			
	*F3H*	Change in the anthocyanin biosynthesis pathway	Klimek-Chodacka et al. (2018)
	*DcMYB113*-like	Anthocyanin biosynthesis	Xu et al. (2019b)
Watermelon			
	*ALS*	Enhanced herbicide resistance	Tian et al. (2018)
Pumpkin			
	*GRF12*, *AHA1*, and *HAK5*	Salt sensitivity	Huang et al. (2019)
Lettuce			
	*LsNCED4*	Seed germination inhibition	Bertier et al. (2018)
Chinese Cabbage			
	*BraFLCs*	The early-flowering phenotype that did not depend on vernalization	Jeong et al. (2019)

### Albino phenotype

Some plants lack chlorophyll pigmentation as a result of phytoene desaturase (PDS) gene disruption, which affects the formation of carotenoids and chlorophyll, leading to albino plant phenotypes. There is little information on the albino plant phenotypes in vegetables except for a few publications where albinism has been used to standardize the gene-editing method utilizing CRISPR-Cas. Fully albino plants (pds mutants) were generated in various vegetable crops like tomato, watermelon, melon, cabbage and carrot *via* editing phytoene desaturase gene through *Agrobacterium tumefaciens*-mediated transformation (Pan et al., 2016; Tian et al., 2017; Xu J. et al., 2019; Hooghvorst et al., 2019; Ma et al., 2019).

## Abiotic stress

Vegetable crops face many abiotic stresses caused due to temperature, drought, salinity and heat which adversely affect crop productivity. Although traditional breeding techniques are able to combat stresses to certain extent, new innovative technologies like CRISPR-Cas 9 offers the possibility to generate more resilient germplasm in dealing with these stresses (Haque et al., 2018). High temperature is a major stress factor that inhibits the growth and productivity of vegetable crops. It leads to the overproduction of reactive oxygen species (ROS) which causes oxidative damage, ultimately impairing the normal function of plant cells. Highly conserved protein kinases called mitogen-activated protein kinases (MAPKs) are involved in the response to heat stress of vegetable crops (Sharma et al., 2020). Knockout of BRASSINAZOLE RESISTANT 1 (BZR1) impaired the induction of RESPIRATORY BURST OXIDASE HOMOLOG1 (RBOH1) and induced production of H_2_O_2_ and heat tolerance in tomato. This exogenous H_2_O_2_ recovered the heat tolerance in bzr1 tomato mutant plants (Yin et al., 2018). In addition to this, mutations induced through CRISPR-Cas9 in *slmapk3* imparts higher heat stress tolerance in tomato. *Slmapk3* mutant also showed less wilting, mild membrane damage, low production of reactive oxygen species and improved antioxidant enzymatic activity under heat stress as reported by Yu et al. (2019). Similarly, in lettuce by using CRISPR/Cas9, LsNCED4 (*9-cis-EPOXYCAROTENOIDDIOXYGENASE4*) gene, resulting in thermo-inhibition of seed germination was knocked out, which significantly resulted in high-temperature germination in both cultivars (Salinas and Cobham Green), capable of germinating more than 70% at 37°C (Bertier et al., 2018).

One of the most harmful environment factors causing damage to vegetable crops is drought stress. Important signalling molecules that react to drought stress include mitogen-activated protein kinases (MAPKs). In 2017, Wang and his co-workers utilized CRISPR/Cas9) mediated mutagenesis to generate slmapk3 mutants in tomato. In comparison to wild type plants, the slmapk3 mutants had more severe wilting symptoms, greater hydrogen peroxide levels, low antioxidant enzyme activity, and experienced more membrane damage. They concluded that slmapk3 is involved in drought response in tomato by protecting from membrane damage and stimulating transcription of some stress related genes. Chilling stress is the primary obstacle that prevents the growth of some vegetable crops, like tomato, brinjal, and chilli as they are sensitive to severe chilling injury. The highly conserved C-repeat binding factors (*CBFs*) are involved in regulating cold tolerance. As in tomato, the slcbf mutants were generated using the CRISPR-Cas9 system, but these mutants exhibited severe chilling- injury symptoms as shown by the down-regulation of CBF-related genes as compared to wild-type (WT) plants (Li et al., 2018a). Additionally in both abovementioned studies, the mutants exhibited lower content of proline and protein and more amount of hydrogen peroxide contents and antioxidants than WT plants which further altered hormonal level of plants and reduced the expression of genes. So, there is a need to study the regulatory mechanism of *CBF* and mitogen-activated protein kinases (MAPKs) genes in order to understand their molecular mechanism.

In addition to this, the SlUVR8 gene was knocked out in tomato to increase tolerance to high UV-B stress using CRISPR-CAS9 gene editing approach by generating sluvr8 mutant lines which confirmed that *SlUVR8* plays a significant function in tomato seedling growth and UV-B stress resistance Liu et al. (2020). Excessive concentration of salts within the plant tissues will reduce growth and productivity, as they can affect several pivotal processes, such as germination, photosynthesis, nutrient balance and redox balance, among others (Parihar et al., 2015; Petretto et al., 2019). Recently, the *HKT1*&*2* allele was edited and inserted into tomato Hongkwang cultivar *via* the CRISPR/Cpf1-mediated homology-directed repair (HDR) mechanism which showed stable inheritance for salt tolerance (Vu et al., 2020). Furthermore, by precise deletion of one or more *SlHyPRP1’s* functional motifs using CRISPR/Cas9-based multiplexed editing, salt stress-tolerant events in cultivated tomatoes were produced (Tran et al., 2021).

### Biotic stresses

Globally, major losses in the production of vegetable crops are caused by a diverse variety of diseases. A sustainable strategy for supplying the world’s expanding population with food is the development of disease-resistant cultivars. (Thomazella et al., 2016). Traditional plant breeding has been utilized for centuries to develop new varieties, but modern technologies, like genome editing, have the ability to produce improved varieties more quickly, by accurately introducing favourable alleles into locally adapted types (Nekrasov et al., 2017). In tomato, *SlDMR6-1* orthologue *Solyc03g080190.2* is up-regulated when infected due to *Pseudomonas syringae* pv. *tomato* and *Phytophthora capsici*. The tomato homologue genes were knocked out using CRISPR-Cas9 to cause mutations in *DMR6*, which resulted in broad-spectrum resistance to *Pseudomonas*, Phytophthora, and Xanthomonas spp. (Thomazella et al., 2016). Wild-type *MILDEW RESISTANT LOCUS O* (*Mlo*) alleles, encode a protein, which provides fungal sensitivity causing powdery mildew disease. In tomato, homozygous loss-of-function of *SlMlo1* gene through CRISPR-mediated mutations resulted in resistance to powdery mildew (Nekrasov et al., 2017).

*Pseudomonas syringae* pv. *tomato (Pto) DC3000*, a causative agent of tomato bacterial speck disease releases coronatine (COR) which stimulates stomatal opening and encourages the bacterial colonization in the leaves. Ortigosa et al. (2019) developed a tomato genotype resistant to bacterial speck by editing the *SlJAZ2* gene (a key co-receptor for coronatine in stomatal guard cells) *via* the CRISPR/Cas9 system to produce dominating Jasmonate-Zim Domain (*JAZ2*) repressors (*SlJAZ2jas*), which prevents coronatine from reopening stomata and provided resistance to PtoDC3000. Jeon et al. (2020) identified biosynthetic gene clusters (*ACET1a, ACET1b* and *Solyc12g100270*) in tomato plants required for the production of falcarindiol in response to biotic stress. Mutagenesis through CRISPR revealed the direct role of the cluster in synthesis of falcarindiol which imparts resistance against fungal and bacterial pathogens in tomato. In 2018, through the use of the CRISPR/Cas9 system, tomato plants were made resistant to the tomato yellow leaf curl virus by focusing Through the use of the CRISPR/Cas9 system, tomato plants were made resistant to the tomato yellow leaf curl virus by focusing on the coat protein and replicase sites (Tashkandi et al., 2018).

In order to speed up the breeding of potatoes for resistance to late blight (Phytophthora infestans) and potato virus Y (PVY), CRISPR/Cas has emerged as a substitute and effective method. Targeting P3, CI, Nib, and CP viral genes, Cas13a protein was used to give resistance to three PVY strains (RNA viruses) (Zhan et al., 2019). Similarly, the functional knockouts of StDND1, StCHL1, DMG400000582 (StDMR6-1) and caffeoyl-CoA-O-methyltransferase gene generated potato plants with increased late blight resistance (Hedge et al., 2021; Kieu et al., 2021). Knocking out of Clpsk1 gene, which encodes the PSK precursor, in watermelon to provide increased resistance to *Fusarium oxysporum* f.sp. *niveum* (Zhang et al., 2020), whereas, in tomatoes, Solyc08g075770-knockout *via* CRISPR-Cas9 resulted *Fusarium* wilt disease sensitivity in the plants (Prihatna et al., 2018). Virus resistance can be induced in cucumber plants by disrupting the function of the recessive eIF4E (eukaryotic translation initiation factor 4E) gene using Cas9/subgenomic RNA (sgRNA) technology (Chandrasekaran et al., 2016). Similarly in *Brassica napus*, BnWRKY11 and BnWRKY70 were edited with CRISPR/Cas9 vectors and mutations induced in BnWRKY70 generated mutants with enhanced resistance to *Sclerotinia* spp. (Sun et al., 2018).

### Vegetable quality improvement

Fruit and vegetables (F&V) are highly perishable food products that need advanced post-harvest technologies to maintain their storage stability and extended shelf life (Gallagher and Mahajan., 2011). In tomato, the homology-directed repair (HDR) pathway was used to replace the allele of ALC with the alc gene, resulting in T1 homozygous plants with long shelf life. (Yu et al., 2017). Klap et al. (2017) used CRISPR/Cas9 technology to delete tomato SlAGL6 (SlAGAMOUS-LIKE6) which led to the development of parthenocarpy under high-temperature stress conditions without compromising the weight, fruit shape, or pollen vitality. Other vegetable crops, like the in-demand seedless watermelon or less-seeded fruits, can also use this approach to generate parthenocarpy.

Lycopene is an important plant nutrient with strong antioxidant properties that helps to protect the cells from damage. Lycopene accumulation in the fruit is facilitated by the knockdown of a few genes linked to the carotenoid metabolic pathway. The amount of lycopene was successfully increased to about 5.1 times in genome-edited tomato fruits. These results suggested that the CRISPR/Cas9 system has the potential to greatly increase the amount of lycopene in tomato fruit due to its high effectiveness, infrequent off-target mutations, and stable heredity Li et al. (2018a). The non-proteinogenic amino acid gamma-aminobutyric acid (GABA) has hypotensive properties. Nonaka et al. (2017) To boost GABA accumulation by 7–15 times in tomato fruits, researchers employed CRISPR/CRISPR-associated protein (Cas)9 technology to remove the autoinhibitory domain of SlGAD2 and SlGAD3 and insert a stop codon right before the autoinhibitory domain.

Potato starch quality is important in various food applications. Improved starch quality with full knockout of granule-bound starch synthase (GBSS), starch synthase gene (*SS6*) and starch-branching enzymes (SBEs) genes SBE1, and SBE2 was reported in potato using CRISPR mediated genome editing (Andersson et al., 2017; Andersson et al., 2018; Kusano et al., 2018; Johansen et al., 2019; Veillet et al., 2019; Sevestre et al., 2020; Zhao et al., 2021). The enzyme polyphenol oxidase (PPO) catalyzes the oxidation of phenolic compounds into highly reactive quinones that cause postharvest browning of cut or bruised fruit (Araji et al., 2014). In the tetraploid potato cultivar Desiree, Gonzalez et al. (2020) investigated the use of the CRISPR/Cas9 system to introduce mutations into the StPPO2 gene. Mutations induced in the four alleles of the *StPPO2* gene led to lines with reduced PPO activity (69%) in tubers. CRISPR/Cas has also been utilized in potato for improving traits like carotenoid biosynthesis (Khromov et al., 2018; Banfalvi et al., 2020; Butler et al., 2020) and glycoalkaloids (Nakayasu et al., 2018). Reducing the amount of steroidal glycoalkaloids (SGAs), such as α-solanine and α -chaconine, in tubers is necessary for breeding excellent potatoes since their presence may give potatoes a bitter flavor and have other unfavorable effects on humans. Two SGA-free potato lines were generated by selectively inhibiting a steroid 16-hydroxylase (St16DOX) that is involved in the synthesis of steroidal glycoalkaloids (SGA) in potato (Nakayasu et al., 2018).

Similarly, in brinjal, the three-polyphenol oxidase (PPO) genes *SmelPPO4*, *SmelPPO5*, and *SmelPPO6* in brinjal were linked to enzymatic browning. To stop the browning of fruit flesh, these three target PPO genes have been eliminated using CRISPR-Cas9-based mutagenesis. (Maioli et al., 2020). It paves the way for the creation of genotypes of eggplant with reduced levels of flesh browning and higher levels of berry polyphenols as this is the first time the CRISPR/Cas9 system has been applied to eggplant for biotechnological uses.

### Yield

Cucumber gynoecious inbred lines are very important because of their better production yield and cheaper labour cost for crossing. Hu et al. (2017) used CRISPR-Cas9 technique to create Cswip1 mutants by targeting the WPP trp/pro/pro domain Interacting Protein1 (CsWIP1) gene, which encodes a zinc-finger transcription factor. Cswip1 T0 mutants had a gynoecious phenotype with exclusively female flowers and these gynoecious mutants will be beneficial in heterosis breeding to produce high-yielding hybrids.

Similarly, Zhang et al. (2019a) created artificial gynoecious watermelon lines by editing *ClWIP1*gene using clustered regularly interspaced short palindromic repeats (CRISPR)/CRISPR-associated system. Additionally*, SP5G* mutations in field tomatoes hasten the blooming process and alter the compact growth habit, resulting in a short flowering interval and an early harvest (Soyk et al., 2017).

### Herbicide resistance

Weeds are a significant stress factor that affects the yield and quality of vegetables, and selective herbicides are frequently used to control the growth and development of weeds during cultivation. The herbicide target gene acetolactate synthase (ALS) has been edited by using CRISPR-Cas9 technology in vegetables like tomato, watermelon, soybean, and potato for developing herbicide resistance in plants (Danilo et al., 2019; Tian et al., 2018; Li et al., 2015; Veillet et al., 2019).

*Phelipanche aegyptiaca*, an obligatory weedy plant parasite, requires the presence of the plant hormone strigolactone (SL) to encourage seed germination. Carotenoid dioxygenase 8 (CCD8), a crucial enzyme in the carotenoid synthesis pathway that generates strigolactone in tomatoes, and More Axillary Growth1 (MAX1), which is involved in strigolactone synthesis, were modified using CRISPR-Cas9 to significantly lower SL content and produce tomato plants resistant to *P. aegyptiaca* (Bari et al., 2019; Bari et al., 2021).

Recently, Yang et al. (2022) designed and tested the efficiency of sgRNA to use with Crispr/Cas system to edit herbicide-related genes *pds* (*phytoene desaturase*), *ALS* (*acetolactate synthase*), and *EPSPS* (*5-Enolpyruvylshikimate-3-phosphate synthase*) in tomato. The outcomes of the sgRNA efficiency tests confirmed that the transformation process could alter the target locations. They verified that 19 different transgenic tomatoes had adequately been edited by ALS2 P or ALS1 W sgRNAs, and 2 of them carried three base mutations that are likely to change their herbicide resistance.

### Regulation of genome-edited crops

Genome/gene editing refers to the precise change in either of DNA or RNA sequence of any target organism. This editing can lead to change in a single base pair to completely reorganization of the large genomic region. Sometimes, genes that are not present in the natural gene pool are also introduced into the target individual to generate novel traits. As this technique involve genetic manipulation either by altering genome sequence or by addition of foreign genomic sequence therefore it becomes mandatory to enforce the regulations of the Cartagena Protocol by any country. The Cartagena Protocol on Biosafety set the foundation for regulating the release and international trade of genetically modified organisms. However, there have been differences in the patterns of GM crop cultivation, utilization and legislation. While some nations restrict production and deny consumption, others actively cultivate and consume them (Garcia Ruiz et al., 2018). Some countries regulate the process while others are involved in the regulation of the product (Eckerstorfer et al., 2019; Van Vu et al., 2019).

For instance, in 2018, as per the guide lines of United States Department of Agriculture (USDA) genome editing through CRISPR-Cas 9 is like conventional breeding therefore does not need any regulation under American Regulatory Standards and are exempted from the regulatory frameworks (Waltz, 2016a). This gives advantage in minimizing the time and resources needed for the testing and legislation of the release of the CRISPR edited crops. Growing research output is authentic evidence that CRISPR-Cas edited crops holds significant promise in improving the yield and quality of crops for the consumers across the world.

In the year 2018, Canadian legislation stated that any gene editing technology which produces a novel product must be subjected to further regulatory supervision on toxicity, allergenicity and any effects on other organisms except the target (Smyth, 2017). For example, non-browning apples and non-dark spot potatoes were approved in Canada after a long examination process which ensured that the changes made in these two products were not harmful to the human being.

However, the European Court of Justice (ECJ) has approved many mutagenic crops developed through chemical and physical mutagens (Waltz, 2016b) but considered gene-edited crops under same strict rules as traditional genetically modified (GM) plants. Among the South American countries like Argentina has developed a regulation system as per the guidelines of Cartagena Protocol on Biosafety for the approval of genome-edited products and relies on the case-by-case evaluation with the exemption from the regulation in the absence of transgene (Whelan and Lema, 2015). Chile and Brazil also followed the same regulatory system regarding genome editing as Argentina. Chile has given regulations in 2017 while Brazil in January 2018 (Duensing et al., 2018).

**FIGURE 4:**
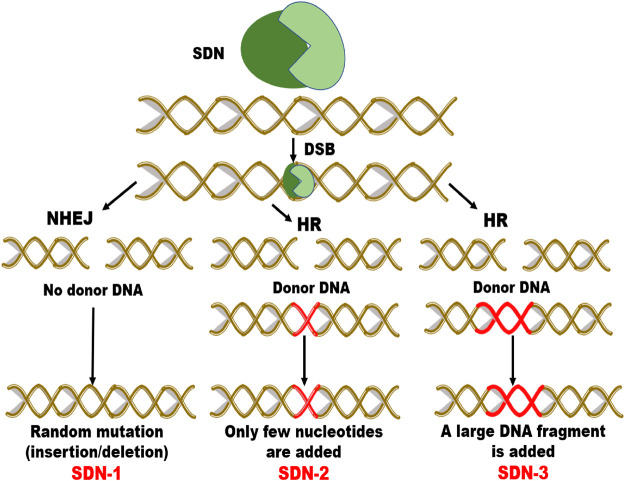
Schematic diagram of modification by site directed nuclease (SDN- 1, SDN-2 and SDN-3) types. Double strand break (DSB) is repaired via non-homologous end joining (NHEJ) or homologous recombination (HR). SDN1 results in random insertion/deletion, SDN2 induces addition of few nucleotides and SDN3 inserts a DNA fragment.

In Australia, the regulatory framework is set by the Gene Technology Act 2000 (GT Act) and GT Regulations 2001 (GT Regulations) with the purpose to protect people’s health, safety and the environment by recognizing threats posed as a consequence of genetic manipulation. The proposed amendments relevant to genome editing would exclude organisms developed with site-directed nucleases (SDN-1) from regulation and stated that organisms developed with SDN2 or SDN-3 are regulated as GMOs (Thysegen, 2019) (Please see Figure 4 depicting mechanism of SDN-1, 2 and 3 types). In the New Zealand, release of genetically modified plants is regulated by the Hazardous Substances and New Organisms (HSNO) Act 1996. The Act generally defines a GMO as any organism whose genome or genetic information has been altered by *in vitro* methods (Fritsche et al., 2018).

In India, all the activities related to the development and use of genetically modified products are regulated as per the ‘‘Rules for the Manufacture/Use/Import/Export and Storage of Hazardous Microorganisms, Genetically Engineered Organisms or Cells’’, 1989 (Rules 1989) which covers new genome editing technologies including CRISPR/Cas9 and notified under the Environment (Protection) Act, 1986, by Genetic Engineering Appraisal Committee (GEAC). (Warrier and Pande 2016). This committee is responsible for granting permits to conduct experimental and large-scale open field trials and also granting approval for the commercial release of genetically altered crops.

### Challenges and future prospects of clustered regularly interspaced short palindromic repeats/CRISPR-associated protein-9 genome editing

The CRISPR/Cas9 system is the latest cutting-edge technology that augments crop improvement by generating high-yielding, better quality, and resistant crop plants to biotic and abiotic stresses crops in a short span of time (Doudna and Charpentier, 2014; Langner et al., 2018). The NHEJ-mediated gene repair creates precise alterations to knock out or change the function of a particular target gene(s) which plays an important role in crop-trait-specific applications, but still there exist many challenges which must be overcome. Foremost is the selection of genes that are to be targeted for mutations and the types of mutation to avoid off-target gene editing. Moreover, it is difficult to carry out genome editing in the target organisms without genome sequencing. Editing a single gene does not result in desired phenotypic changes, because significant agronomic factors are quantitative. To add desired mutant alleles, effective CRISPR-Cas-mediated target site-specific insertion, deletion, and chromosomal recombination procedures can be applied (Zhu et al., 2020).

Once a gene has been identified, the second major challenge is to deliver CRISPR-Cas gene-editing agents into plant cells and the procedure to regenerate the putative edited plants (Chuang et al., 2021; Lino et al., 2018). Actually, it is quite challenging to create a universal and effective genetic transformation and regeneration system for vegetable crops (Niazian et al., 2017). In addition to this, for successful genetic transformation of vegetable crops editing efficiency is to be considered, which is further influenced by various factors, such as the number of sgRNA and GC amount; the expression levels of sgRNA and Cas9; and the secondary structure of the paired sgRNA and target sequence. (Kumlehn et al., 2018; Hu et al., 2019). Since genome editing in vegetable crops has such huge potential, we expect that strategies will be formulated to overcome these challenges in the near future.
